# Preparation and Characterization of Electrospun PLCL/Poloxamer Nanofibers and Dextran/Gelatin Hydrogels for Skin Tissue Engineering

**DOI:** 10.1371/journal.pone.0112885

**Published:** 2014-11-18

**Authors:** Jian-feng Pan, Ning-hua Liu, Hui Sun, Feng Xu

**Affiliations:** 1 Department of Orthopaedics, Shanghai Jiao Tong University Affiliated Sixth People's Hospital, Shanghai, China; 2 Department of Orthopaedics, Kunshan Traditional Chinese Medical Hospital, Suzhou, Jiangsu, China; Texas A&M University Baylor College of Dentistry, United States of America

## Abstract

In this study, two different biomaterials were fabricated and their potential use as a bilayer scaffold for skin tissue engineering applications was assessed. The upper layer biomaterial was a Poly(ε-caprolactone-co-lactide)/Poloxamer (PLCL/Poloxamer) nanofiber membrane fabricated using electrospinning technology. The PLCL/Poloxamer nanofibers (PLCL/Poloxamer, 9/1) exhibited strong mechanical properties (stress/strain values of 9.37±0.38 MPa/187.43±10.66%) and good biocompatibility to support adipose-derived stem cells proliferation. The lower layer biomaterial was a hydrogel composed of 10% dextran and 20% gelatin without the addition of a chemical crosslinking agent. The 5/5 dextran/gelatin hydrogel displayed high swelling property, good compressive strength, capacity to present more than 3 weeks and was able to support cells proliferation. A bilayer scaffold was fabricated using these two materials by underlaying the nanofibers and casting hydrogel to mimic the structure and biological function of native skin tissue. The upper layer membrane provided mechanical support in the scaffold and the lower layer hydrogel provided adequate space to allow cells to proliferate and generate extracellular matrix. The biocompatibility of bilayer scaffold was preliminarily investigated to assess the potential cytotoxicity. The results show that cell viability had not been affected when cocultured with bilayer scaffold. As a consequence, the bilayer scaffold composed of PLCL/Poloxamer nanofibers and dextran/gelatin hydrogels is biocompatible and possesses its potentially high application prospect in the field of skin tissue engineering.

## Introduction

Adult skin consists of two tissue layers: a keratinized stratified epidermis and an underlying thick layer of collagen-rich dermal connective tissue providing support and nourishment. Because the skin serves as a protective barrier against the outside world, any break in it must be rapidly and efficiently mended [1]. Full thickness grafts, consisting of the epidermis and the full thickness of the dermis, are commonly used in plastic and reconstructive surgery [2]. However, the donor site following the harvest of a full thickness graft has no epidermal elements from which new skin can regenerate. So the grafts must be taken from sites of the body where the donor defects can be primarily closed. This limits the harvest of full thickness grafts clinically. Tissue-engineered skin replacements such as cultured autologous and allogenic keratinocytes grafts, autologous or allogenic composites, acellular biological matrices, and cellular matrices including biological substances such as fibrin sealant and various types of collagen and hyaluronic acid (HA) have opened new options to treat such massive skin loss [3].

Electrospinning is an effective technique to produce polymer nanofibers. It involves using a strong electrical field to rapidly stretch a polymer solution into fine filaments. The solvent evaporation from the filaments leads to the formation of dry or semi-dry fibres, which deposit randomly on the collector forming a nonwoven mat in most cases. In previous studies, synthetic biodegradable polymers, such as poly (lactic acid) (PLA) [4], poly (glycolic acid) (PGA) [5], poly (lactide-co-glycolide) (PLGA) [6], poly (e-caprolactone) (PCL) [7], poly (glycolide-co-caprolactone) (PGCL) [8] and poly (L-lactide-co-e-caprolactone) (PLCL) [9]–[10] were developed for vessel or skin tissue engineering. Among them, PLCL is well known as an elastic biodegradable material; therefore, it was applied as tissue engineering scaffolds to mimic the natural stratified epidermis. However, the hydration and degradation of PLCL contributes to an acidic microenvironment, which is not in favor of the skin regeneration. Poloxamers are non-ionic surfactants and have wide-ranging applications in various biomedical fields including drug delivery and medical imaging [11]–[12]. With use of PLCL/poloxamer blended nanofibers the formation of acidic microenvironment associated with PLCL degradation was prevented as poloxamer neutralizes the production of lactic acid during PLCL degradation in the body.

Underlying the epidermis is thick layer of collagen-rich dermis. It provides physical strength and flexibility to the skin, as well as being the matrix that supports the extensive vasculature, lymphatic system and nerve bundles. The dermis is relatively acellular, being composed predominantly of an extracellular matrix (ECM) of interwoven collagen fibrils. Gelatin, a collagen-hydrolyzed protein with unique gelation behavior under room temperature has been widely researched in food and pharmaceutical industries [13]–[15]. Based on previous studies, by altering the carboxyl groups to amino groups through the reaction with ethane diamine, gelatin could stay liquid state at room temperature [16]–[17]. While dextran was oxidized by sodium periodate to obtain aldehyde groups, the dextran could react with gelatin to form a hydrogel filling in the dermis defect area. Therefore, we fabricated a bilayer scaffold composed of a PLCL/Poloxamer nanofiber upper layer and a dextran/gelatin hydrogel sublayer to mimic the physical structure of the normal skin more accurately.

In this study, the physical properties, mechanical strength and biocompatibility of two separate biomaterials were investigated. For electrospun PLCL/poloxamer nanofibers, morphological characterization was determined through SEM and hydrophilicity was assessed with water contact angle method by a drop shape analysis system. Then the mechanical performance was examined to determine whether the samples can withstand the applied stress. For dextran/gelatin hydrogels, biodegradation was investigated by immersing the samples in PBS for 21 days to determine weight loss over time. The water absorption property of hydrogels was determined by swelling tests after 24 hours of incubation in phosphate buffered saline (PBS). Cell proliferation and viability tests were done to evaluate the biocompatibility of the scaffolds in vitro. The results of the tests performed determined which conditions were optimal to construct the bilayer scaffold that will be used for skin tissue engineering applications.

## Materials and Methods

### Materials

PLCL was purchased from DaiGang biomaterial Co., Ltd. (ShanDong, China). Poloxamer, dextran, N-(3-Dimethylaminopropyl)-N′-ethylcarbodiimide hydrochloride crystalline (EDC), gelatin, sodium periodate, Tetrahydrofuran (THF), ethylenediamine(ED) and N, N-dimethylformamide (DMF) were purchased from Sigma-Aldrich (St Louis, MO, USA). Fetal bovine serum (FBS), phosphate buffered saline (PBS), Dulbecco's modified Eagle's medium (DMEM), Live/Dead Viability Assay Kit, collagenase II, penicillin-streptomycin solution, trypsin-EDTA and other culture media and reagents were purchased from Gibco Life Technologies Corporation (Carlsbad, CA, USA). CCK-8 was purchased from Dojindo Corporation (Kumamoto, Japan). Tissue culture flasks were obtained from BD Biosciences Corporation (San Jose, CA, USA). Mouse pre-osteoblast cells (MC3T3-E1) were obtained from the institute of Biochemistry and Cell biology (Chinese Academy of Sciences, China).

### Preparation of scaffolds

#### 1. Electrospun PLCL/Poloxamer membranes

The mixed solvent of THF and DMF (v/v  = 1/1) was used to prepare the electrospinning solutions at a polymer concentration of 8 wt%. In order to investigate the hydrophilicity enhancement of Poloxamer on PLCL fibers, two different compositions of PLCL and Poloxamer mixtures (9/1, 3/1, w/w) were prepared. The electrospinning solution was ejected at a speed of 1.0 mL/h under a fixed electrical potential of 16 kV with a distance of 20 cm between tip of the needle and the collector. All electrospun fibers were deposited on a rotating collector consists of aluminum foil to form a thin fibrous membrane. The fibrous mats were placed in vacuum drying at room temperature to completely remove any solvent residue.

#### 2. Dextran/gelatin hydrogels

Dextran was oxidized by reacting with sodium periodate as reported. Briefly, dextran solution was prepared by dissolving 10 g of dextran in 100 ml of distilled water. 6.34 g of NaIO_4_ (dissolved in 100 ml of distilled water) was added dropwise to the dextran solution. The solution was stirred at room temperature for 6 hours and shielded from light. Then 2 ml of ethylene glycol was added to terminate the oxidation reaction. The resulting solution was dialyzed exhaustively for 3 days against water and lyophilized to obtain the final dextran.

The carboxyl groups in gelatin were converted into amino groups by reaction with ED in the presence of EDC. Gelatin was dissolved in 100 ml of phosphate buffered solution (PBS) to a final concentration of 5 wt% at room temperature and 16 ml of ethylenediamine was added. Immediately after that, the pH of solution was adjusted to 5.0 by adding hydrochloric acid (HCl). After that 2.3 g of EDC was added into the gelatin solution. The molar ratio of the carboxyl groups on gelatin chains, EDC and ED was 1∶2∶40. The reaction mixture was stirred at room temperature overnight, and then dialyzed against distilled water for 48 hours to remove the excess ED and EDC. The dialyzed solution was freeze-dried at −80°C to obtain a modified gelatin.

Dextran was dissolved in PBS to achieve a concentration of 10% (wt/vol%) and gelatin was diluted to achieve a 20% (wt/vol%) solution. While the two solutions were mixed together, the hydrogels were formed rapidly through a Schiff-base reaction between aldehyde groups and amino groups. The mixture solution was injected into round molds and then incubated at 37°C for gel forming.

#### 3. Bilayer scaffold

Following the characterization of the electrospun PLCL/Poloxamer nanofibers and dextran/gelatin hydrogels, the 9/1 PLCL/Poloxamer nanofiber membrane and 5/5 dextran/gelatin hydrogel were shown to display favorable physical properties and cell-material interactions [18]–[19]. A bilayer scaffold was fabricated using these two materials by underlaying and casting method. 9/1 PLCL/Poloxamer nanofiber membrane was fabricated and was laid in a 6-well tissue culture plate. Then 5 ml of dextran/gelatin solution at the ratio of 5/5 was poured on the surface of 9/1 PLCL/Poloxamer membrane to form hydrogel and get bilayer scaffolds.

### Characterization of electrospun PLCL/Poloxamer membranes

#### 1. Fiber size analysis

To evaluate the morphology and fiber diameters of electrospun fibers, materials were gold-coated and observed using scanning electron microscope (SEM, JSM-5600LV, JEOL, Japan) at an accelerating voltage of 20 kV. For each sample (n = 3), five random spots were captured to generate micrographs, and at least 20 different fibers were randomLy selected for further measurement using ImageJ software, version 1.46r.

#### 2. Pore Size Measurements

A CFP-1100-AI capillary flow porometer (PMI Porous Materials Int. USA) was used in this study to measure the pore size. Galwick with a defined surface tension of 21 dynes cm^−1^ (PMI Porous Materials Int. USA) was used as the wetting agent for porometry measurements. Electrospun fibrous scaffolds were cut into 3×3 cm^2^ squares and then soaked into the wetting agent. The soaked scaffolds were placed in adapting pan and sealed with O-rings for porometry measurement.

#### 3. Tensile test

To ensure the mechanical properties of fibrous mats falls in the physiological range of human skin, mats were placed in phosphate buffered saline (PBS, Gibco, Invitrogen, USA) for 30 min and subsequently conducted following standard mechanical test. The fabric materials (200 µm in thickness) were punched into rectangular strips (70 mm×7 mm, n = 5) and characterized by a tensile test (Instron 5567, Canton, MA). The stress-strain curves of these materials were constructed from the load-deformation curves recorded at a stretching speed of 0.5 mm/s. Ultimately the tensile strength, Young's modulus and elongation at break were obtained from plotted stress–strain curves. Tensile property values reported here represent an average of the results for tests run on at least five samples.

#### 4. Measurement of water contact angle

To determine the influence of poloxamer on the hydrophilicity of PLCL, water contact angle test was measured using a commercial drop shape analysis system (Data Physics SCA20, Germany). The fabric materials were cut into pieces approximately 1×1 cm (n = 6) and air-dried at room temperature for 48 h, then 3 µL deionized droplets were gently deposited on each sample through a micro syringe, images were captured at 2 s after the water droplet was dripped on the surface of materials, and the contact angle was measured by the inbuilt software in the machine.

### Characterization of dextran/gelatin hydrogels

#### 1. Swelling analysis

To study the swelling kinetics of the modified hydrogels, the dextran/gelatin hydrogels at different ratios (3/7, 4/6, 5/5, 6/4, 7/3) were frozen at −80°C and lyophilized in a vacuum oven. The dry hydrogels were weighed (W_d_) and then immersed in PBS at 37°C. After 24 h of incubation, the samples were removed from the PBS and the water on the surface was quickly wiped out with a filter paper so that the swollen weight could be measured (W_s_) accurately. The swelling ratio (SR) was then calculated according to the following equation: SR = W_s_/W_d_.

#### 2. In vitro degradation

To determine the weight loss due to hydrolytic degradation, hydrogels were divided into four groups (day 3, day 7, day 14, day 21) for time-control degradation study. The samples (n = 3) were prepared from blending 10 wt% dextran and 20 wt% gelatin aqueous solutions in different ratios (3/7, 4/6, 5/5, 6/4, 7/3) and pre-swollen in PBS overnight. Subsequently, the weight of the sample was record as W_0_ and the hydrogels were completely submerged in PBS at 37°C. At different time intervals, the samples were removed from the solution, blotted dry and then weighed to determine the weight of the remaining mass (W_1_). The PBS was replaced every 3 days and the experiments were performed in triplicate. The weight loss (%) was calculated as the following formula: weight loss  =  (W_0_–W_1_)/W_0_×100%.

#### 3. Compression test

The hydrogels were also characterized by compression stress-strain measurements using a Dejie DXLL-20000 materials testing instrument at 25°C. Based on the early screening studies, samples were incubated in PBS at 37°C for 24 h before test in order to reach completely swelling equilibrium. After measuring diameter and thickness of the specimens, they were put on the lower plate and compressed by the upper plate at a strain rate of 1 mm/min. The initial compressive modulus was determined by the average slope in a range of 0–10% strain from the stress-strain curves. The fracture stress, determined from the peak of the stress-strain curve, was also reported. All compression testing groups had a sample quantity of n = 3.

### Biological assess of electrospun PLCL/Poloxamer membranes and dextran/gelatin hydrogels

#### 1. Cell isolation and culture

Animal procedures related to adipose tissue isolation were approved by the Shanghai JiaoTong University Ethical Committee. After the 10% chloral hydrate (350 mg/kg) anesthesia of rats, abdominal adipose tissue (approximately 5 g) was obtained from bilateral inguinal region of SD rats and washed with PBS for 15 min. Tissue was minced by sharp dissection into 1 mm^3^ pieces, and directly exposed to PBS containing 0.1% collagenase type I (Sigma–Aldrich, St. Louis, MO) for enzymatic digestion. After 60 min incubation at 37°C with mild agitation (40 rpm), an equal volume of Dulbecco's modified Eagle's medium (DMEM, Gibco) containing 10% FBS was added to stop enzymatic digestion. Then the mixed solution was filtered through a 70 µm nylon mesh and the filter liquor was transferred into a 15 mL centrifuge tube, finally the cellular pellet was isolated via centrifugation 1500 rpm for 10 min at room temperature. Cells were dispensed into tissue culture flasks (Corning Glass Works, Corning, NY) containing 5 mL complete medium. ADSCs were incubated in a 5% CO_2_ incubator at 37°C, and medium was changed every 3 days.

#### 2. Cell viability assay of electrospun PLCL/Poloxamer membranes

Cell viability was determined using a CCK-8 Assay Kit. Electrospun matrices were cut into 12 mm diameter circles and sterilized by immersion in 70% ethanol for 1 h, subsequently they were washed with PBS (supplemented with 500 U/mL penicillin and 500 U/mL streptomycin) and DMEM three times separately. ADSCs were suspended in complete medium and seeded with a density of 5×10^3^ cells per each sample, and they were also seeded on tissue culture plate (TCP) as a control group. Then the 24-well tissue culture plate was incubated under a humidified atmosphere of 5% CO_2_ at 37°C. The culture medium was exchanged every day. After 1, 3 and 7 days culture, 400 µl CCK-8 mixed solution (400 µl medium containing 40 µl CCK-8 reaction solution) was added to each well and incubated for 2 hours at 37°C. Then the medium with CCK-8 was transferred to 96-well tissue culture plate and the absorbance was read at 450 nm. All experiments were carried out in triplicate.

#### 3. Cell viability assay of dextran/gelatin hydrogels

For this assay, each 300 µl mixed solution was injected into 24-well tissue culture plate and formed hydrogel at 37°C. Cell suspension with cell density of 1×10^6^ cells/mL was injected on the surface of hydrogel. The cell-seeding hydrogels were incubated at 37°C in a humidified atmosphere of 5% CO_2_. The culture medium was exchanged every day. After 1, 3 and 7 days culture, 400 µl CCK-8 mixed solution (400 µl medium containing 40 µl CCK-8 reaction solution) was added to each well and incubated for 2 hours at 37°C. Then the medium with CCK-8 was transferred to 96-well tissue culture plate and the absorbance was read at 450 nm. All experiments were carried out in triplicate.

#### 4. Cytotoxicity assay of bilayer scaffold

The cytotoxicity assay of bilayer scaffold was quantified using the CCK-8 assay for 1, 3, 7 and 14 days through a Transwell system (Costar 3422). Briefly, the adipose-derived stem cells suspension with cell density of 1×10^6^ cells/mL was injected in 24-well culture plates. The bilayer scaffold was placed within the upper chambers and the upper chambers were inserted in 24-well culture plates. ADSCs seeded in 24-well culture plate without upper chamber served as control group. The complete medium was added and culture plates were incubated at 37°C in a humidified atmosphere of 5% CO_2_. The culture medium was exchanged every three days. After 1, 3, 7 and 14 days culture, the culture medium was removed and 400 µl medium containing 40 µl CCK-8 reaction solution was added to each well and incubated for 4 hours at 37°C and 5% CO_2_. Then the medium with CCK-8 was transferred to 96-well tissue culture plate and the absorbance was read at 450 nm using a multidetection microplate reader (MK3, Thermo, USA). All experiments were carried out in triplicate.

### Statistical Analysis

Values are expressed as mean ± SD. One-way analysis of variance (ANOVA) was used to discern the statistical difference between groups. Repeated-measures analysis of variance (rMANOVA) and Least-significant difference (LSD) were utilized to compare the between-subjects effects of time and group. For all analyses, a two-tailed P value of less than 0.05 indicated statistical significance. Statistical analysis was conducted using SPSS 16.0 for Windows (SPSS, Chicago, USA).

## Results

### Electrospun PLCL/Poloxamer membranes

#### 1. Morphology of PLCL and PLCL/Poloxamer nanofibers

**Figure 1:**
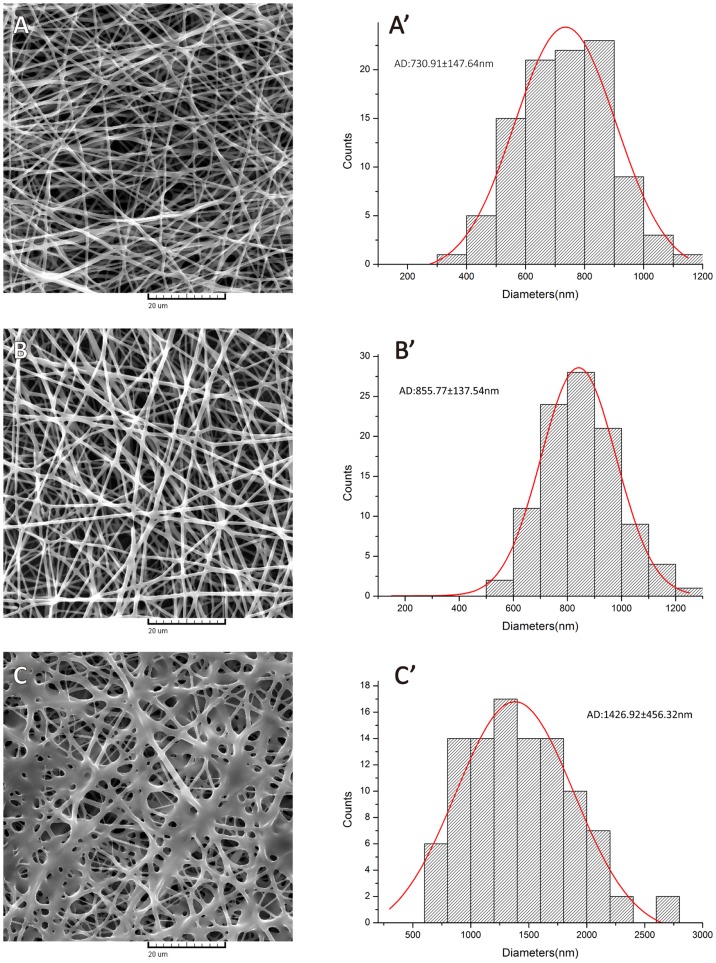
SEM images of electrospun PLCL/Poloxamer nanofibers with different weight ratio of PLCL to Poloxamer: (A) 1/0; (B) 9/1; (C) 3/1.

The SEM morphologies of PLCL and PLCL/Poloxamer nanofibers were shown in Figure 1. Smooth surface and interconnected porous structures of PLCL and PLCL/Poloxamer nanofibers have been obtained. From the micrographs, it is clear that the ratio of PLCL/Poloxamer significantly affected the fiber diameter distributions. The average diameter of PLCL nanofibers from 8 wt% solution is 730.91±147.64 nm, while PLCL/Poloxamer nanofibers of 9/1 and 3/1 have the average diameters of 855.77±137.54 nm and 1426.92±456.32 nm respectively. Fiber average diameters gradually increased with increasing Poloxamer content. At the same time, an enhanced non-uniformity and non-homogeneity of fibers was also noted as the Poloxamer increased.

#### 2. Pore Diameter Analyses

Microscale and nanoscale porous structure of electrospun nanofibrous scaffolds play critical roles for cellular growth and tissue regeneration because the highly porous network of interconnected pores provides nutrients and gas exchange for cell proliferation. SEM showed that the nanofibers had a solid surface with interconnected voids, so that a porous structure was present. Pore diameters of PLCL and PLCL/Poloxamer nanofibers were shown and summarized in Table 1. When blended ratios ranged from 1/0 to 9/1 mean pore diameter increased with increasing the content of Poloxamer. According to a published report [20], as expected the fiber diameter increased, the average pore size of the scaffolds increased. With increasing the content of Poloxamer, the fiber diameter increased so that the pore diameter increased. However, mean pore diameter of 3/1 PLCL/Poloxamer nanofibers was smaller than that of 9/1 PLCL/Poloxamer nanofibers. This may be caused the fact that a large amount of fibers accumulated disorderly and bonded together in 3/1 PLCL/Poloxamer nanofibers showed in Figure 1. Therefore, the pore structure of scaffolds might be jammed with increasing bonded fibers. As expected that cells infiltrated the scaffolds with lager pore diameters and the nanofibrous scaffold with small pore diameter exhibited reduced cellular infiltration. So the 9/1 PLCL/Poloxamer nanofibers might mimic the native ECM and promote cell more spreading in skin tissue engineering.

**Table 1:** Pore diameter of PLCL and PLCL/Poloxamer nanofibers with various blend ratios.

PLCL/Poloxamer ratio	Specimen thickness (mm)	Mean pore diameter ± SD (µm)	Largest pore diameter (µm)	Smallest pore diameter (µm)
1/0	0.07	1.73±0.44	2.85	0.96
9/1	0.05	2.05±0.39	3.03	1.01
3/1	0.10	1.57±0.65	2.60	0.92

#### 3. Water contact angle assay

**Figure 2:**
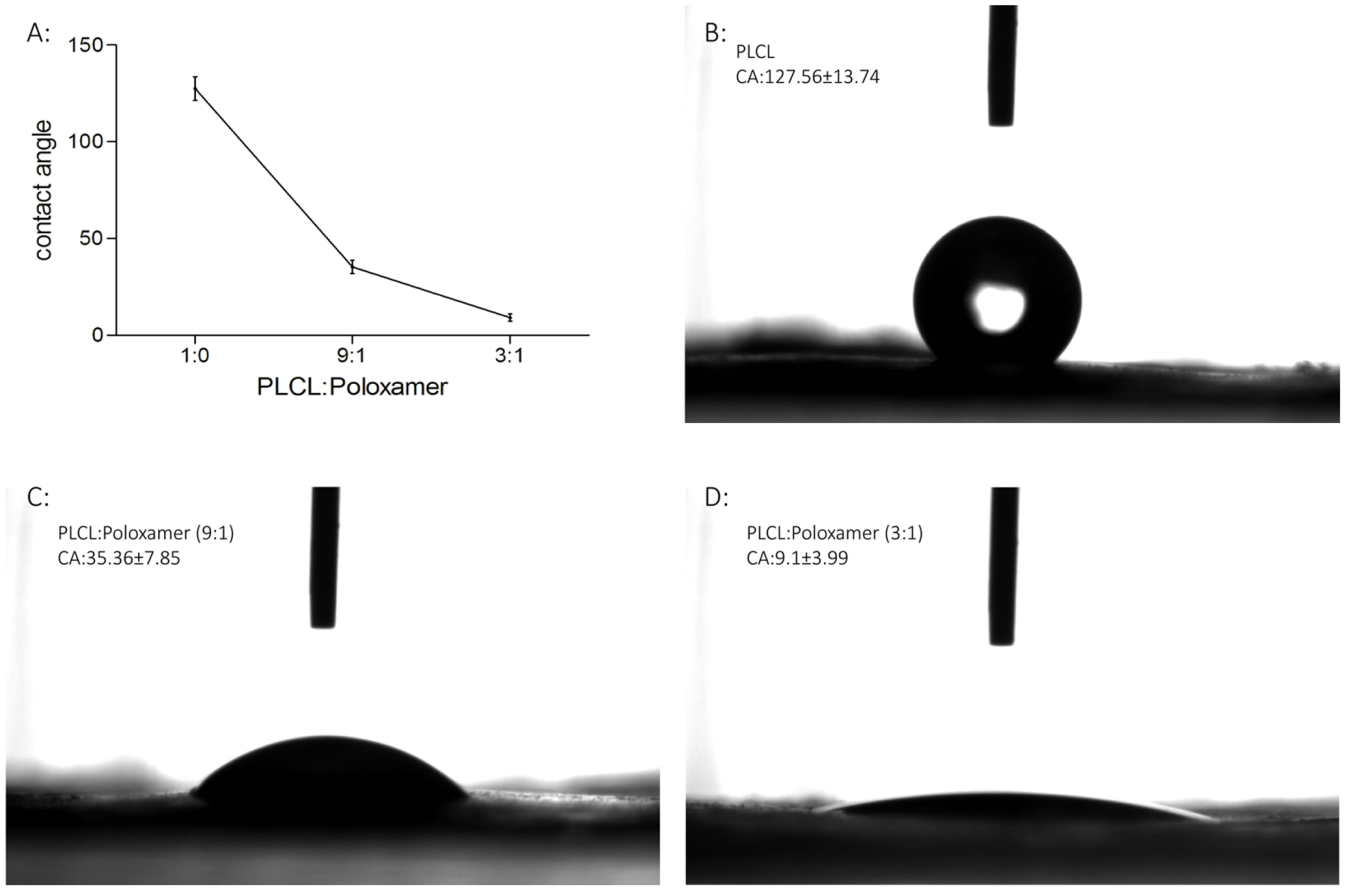
Digital pictures of water contact angles of electrospun PLCL/Poloxamer nanofibers with different weight ratio of PLCL to Poloxamer: (B) 1/0; (C) 9/1; (D) 3/1.

The surface hydrophilic property plays an important role in cell adhesion, spreading, and proliferation on the biomaterials surfaces. To investigate the influence of different blending ratios on the surface hydrophilic property of electrospun PLCL and PLCL/Poloxamer nanofibers, the water contact angle measurement was done and shown in Figure 2. The pure PLCL nanofibers showed a contact angle of about 127.56°±13.74°, indicating that the surface was hydrophobic. In contrast, the electrospun PLCL/Poloxamer nanofibers exhibited more hydrophilic properties, which have an apparent decrease in contact angle. As the Poloxamer content increased, the contact angle of blended nanofibers decreased to approximately 0°. Therefore, the surface wettability of hybrid nanofibers can be obtained by introducing Poloxamer in the blended PLCL/Poloxamer nanofibers.

#### 4. Mechanical properties

**Figure 3:**
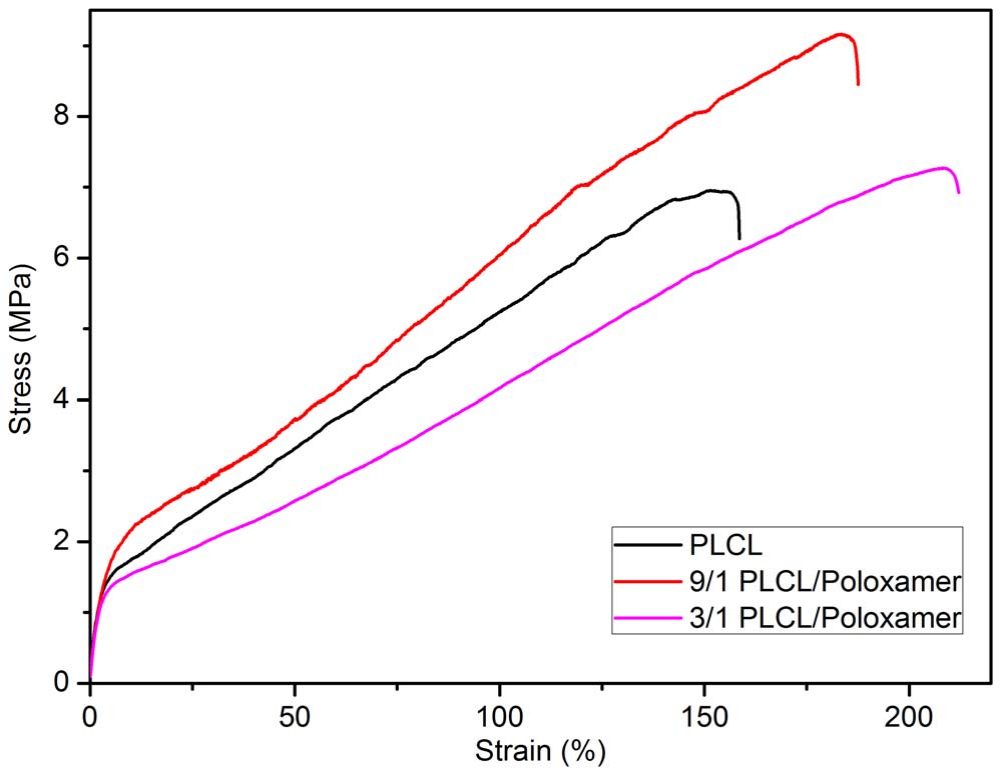
Stress-strain curves for electrospun PLCL/Poloxamer nanofibers with different weight ratio of PLCL to Poloxamer.

Mechanical properties of PLCL/Poloxamer nanofibers are critical for their successful application in skin tissue engineering. For example, a major cause of graft failure in skin substitutes is ischemic and nutrient-deprived, which is often caused by the compliance mismatch between the graft and the host skin tissue [21]–[22]. Therefore, the appropriate mechanical compatibility between PLCL/Poloxamer nanofibers and host skin tissues is a requirement for functioning soft tissue substitutes. Tensile tests were performed on all scaffolds to determine whether the tensile strength properties were favorable for use as a skin graft. Figure 3 shows the typical tensile stress–strain curve of electrospun PLCL and PLCL/Poloxamer nanofibers. The ultimate tensile strength, tensile modulus and elongation at break were summarized in Table 2. The tensile strength and modulus of nanofiber substrates lie well within the range of those of human skin. It is desirable that the tensile properties are similar to those of human skin, providing it with good resilience and compliance to movement as a skin graft. On the other hand, the nanofibrous scaffolds have a larger ultimate strain compared with human skin. This reinforces its potential as a skin graft, since it could still cover the wound when immobilized at a wound site under a high tensile strength. It can also be observed that the PLCL/Poloxamer nanofibers showed higher tensile strength and ultimate strain than PLCL nanofibers. Moreover, the average tensile strength of PLCL/Poloxamer nanofibers (9/1) was 9.37±0.38 MPa with an ultimate strain of 187.43±10.66% when compared with 7.23±0.16 MPa and 158.54±6.67% for PLCL nanofibers, 7.85±0.65 MPa and 215.23±16.41% for PLCL/Poloxamer nanofibers(3/1). It indicates that the introduction of hydrophilic Poloxamer can improve the electrospinnability, thus forming nanofibers with solid surface and interconnected structures which can be benefit for the enhancement of mechanical properties. Thus, it is possible to create hybrid scaffolds with desirable mechanical property for engineering of various soft tissues by selecting the optimum blend ratios of two components. The blended PLCL/Poloxamer nanofibers with PLCL/Poloxamer ratio of 9/1 were selected for further studies as they exhibited better comprehensive properties including hydrophilicity, pore diameter and mechanical strength than other ones.

**Table 2:** Mechanical properties of PLCL and PLCL/Poloxamer nanofibers at various blend ratios compared with human skin.

	PLCL/Poloxamer			
Property	1/0	9/1	3/1	Human skin
Tensile strength (MPa)	7.23±0.16	9.37±0.38	7.85±0.65	5–30
Tensile modulus (MPa)	47.65±2.24	47.49±5.44	46.86±2.54	15–150
Elongation at break (%)	158.54±6.67	187.43±10.66	215.23±16.41	35–115

Values represent the average ± standard deviation.

### Dextran/gelatin hydrogels

#### 1. Morphology

**Figure 4:**
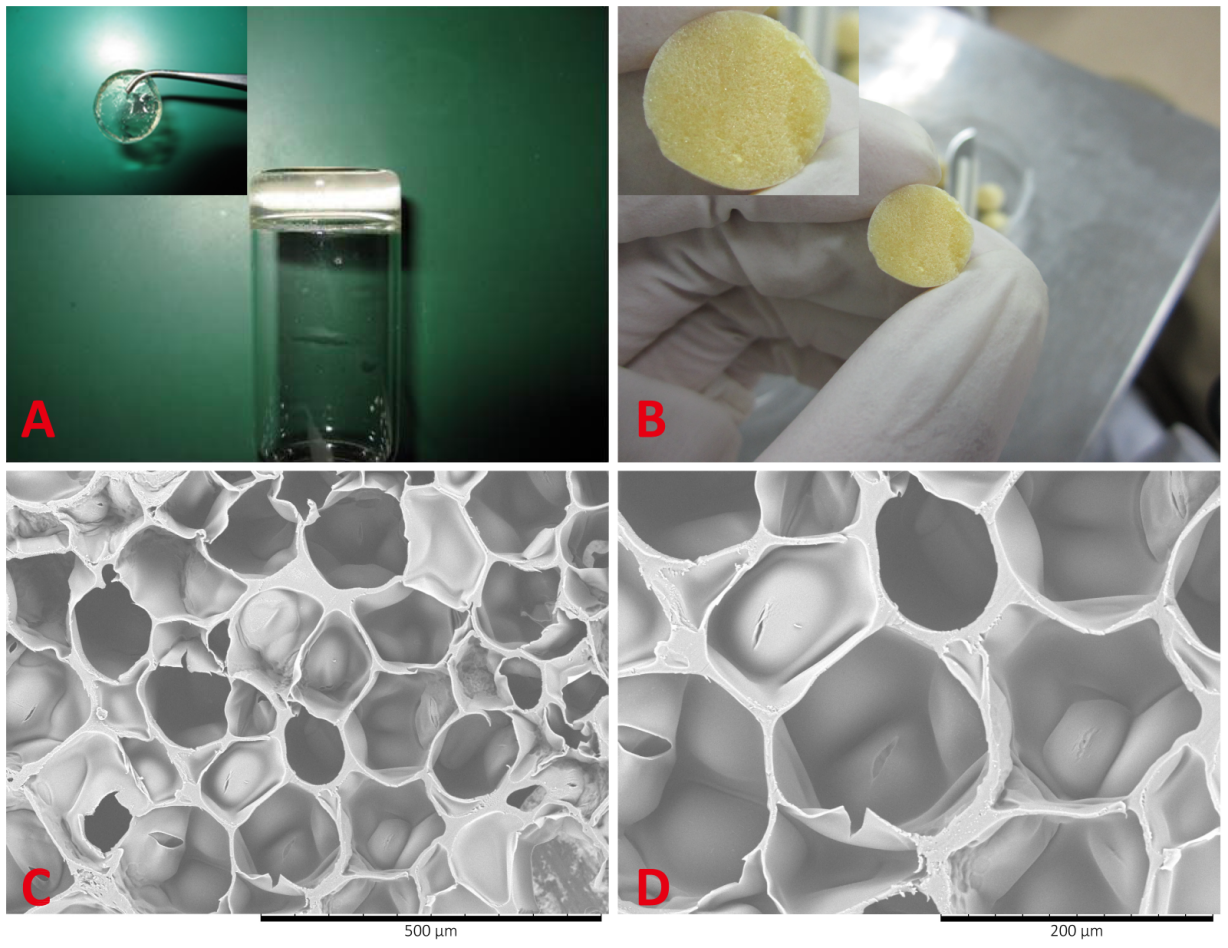
Morphology of the dextran/gelatin hydrogels. A: Gross view of the dextran/gelatin hydrogel; B: Gross view of lyophilized dextran/gelatin hydrogel; C: SEM micrograph of lyophilized dextran/gelatin hydrogel. Scale bar represents 500 µm. D: SEM micrograph of lyophilized dextran/gelatin hydrogel. Scale bar represents 200 µm.

As shown in Figure 4A, dextran and gelatin solutions were mixed and the gel can be formed in a short time. The dextran/gelatin hydrogel was transparent and yellowish in color. In this study, all hydrogel samples were flash-frozen at −80°C in liquid nitrogen and lyophilized. After that, the corresponding cross-section of the dry samples was observed and the freeze-dried hydrogel was yellowish and porous (Figure 4B). Figure 4C and 4D showed the SEM micrographs of freeze-dried hydrogels. All hydrogels showed good interconnected porous structures with an average pore sizes ranging from approximately 50–200 µm. The interconnected porous structure is necessary for scaffolds to promote nutrient and gas diffusion, allow cellular ingrowth and retain high water. Therefore, the hydrogels might be suitable as carriers for cell delivery in skin tissue engineering.

#### 2. Swelling analysis

**Figure 5:**
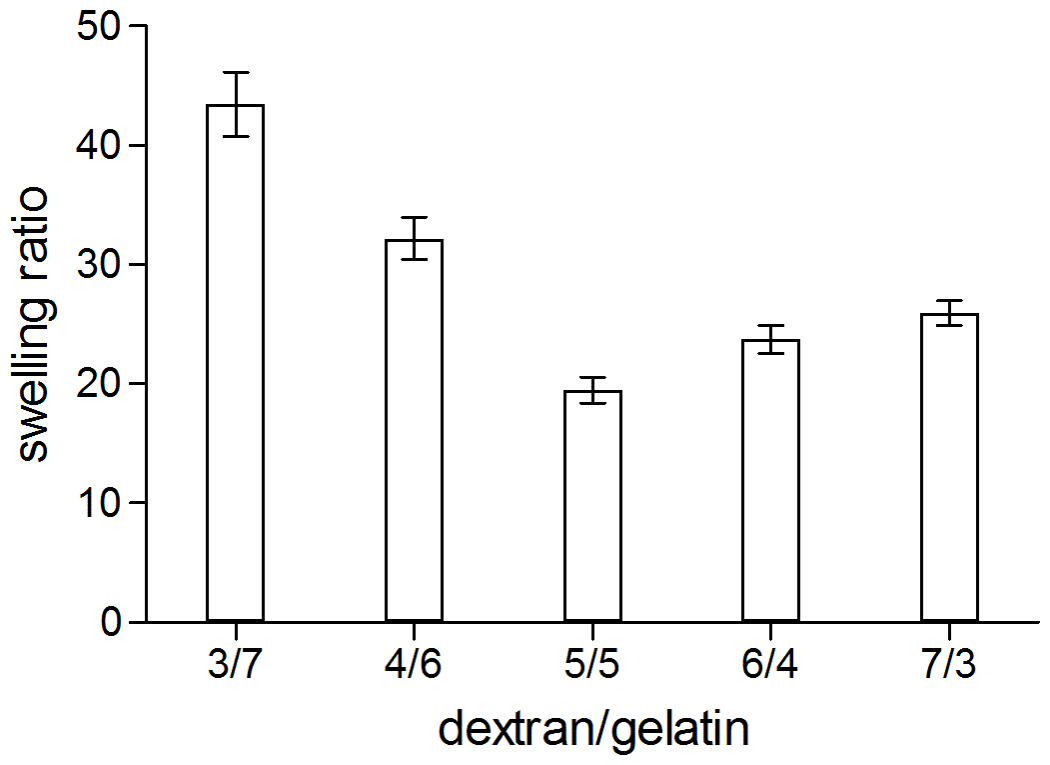
The swelling ratio of dextran/gelatin hydrogels with different volume ratio.

The swelling behavior is an important property of tissue engineering scaffold because it relates to the diffusion of signaling molecules and nutrients. Figure 5 indicates the swelling results of freeze-dried dextran/gelatin hydrogels in PBS. The dry hydrogels could absorb large quantity of water from 19.47 to 43.45 times of their original dry weight, suggesting that they could be good scaffolds to retain tissue fluid and nutrients in vivo. Figure 5 also revealed the relationship between the swelling ratio of the dextran/gelatin hydrogels and the dextran content in the hydrogels. Generally, the swelling ratio of a hydrogel is related to physic-chemical factors, such as the crosslinking density, gel composition, network structure, etc.

The swelling ratio decreased rapidly from 43.45 to 19.47 with the increase of dextran's content from 30 to 50%, which can be ascribed to the increase of crosslinking degree. Thereafter, with an elevation of dextran's content from 50 to 70%, the crosslinking density in the hydrogels declined and the swelling ratio increased slightly from 19.47 to 25.94. In this regard, the variation of the swelling ratio corresponds well with the information of the gelation time depicted in Figure 3. It is also worthwhile to notice that group 5/5 exhibited apparently different swelling property compared with groups of 3/7 and 4/6. We ascribe this obvious decline on swelling ratio to the decrease content of gelatin, which have a strong ability to absorb water. From the clinical aspect, over load swelling ratio of the scaffolds may cause pressure to the surrounding tissues. In contrast, under load swelling ratio of the scaffolds would result in insufficient nutrients exchanged from surrounding circumstances. Meanwhile, the scaffolds with inadequate swelling ratio may escape from the implant point easily. Therefore, the dextran/gelatin hydrogel with an adjustable swelling property can meet those requirements by changing the ratio of components.

#### 3. In vitro degradation

**Figure 6:**
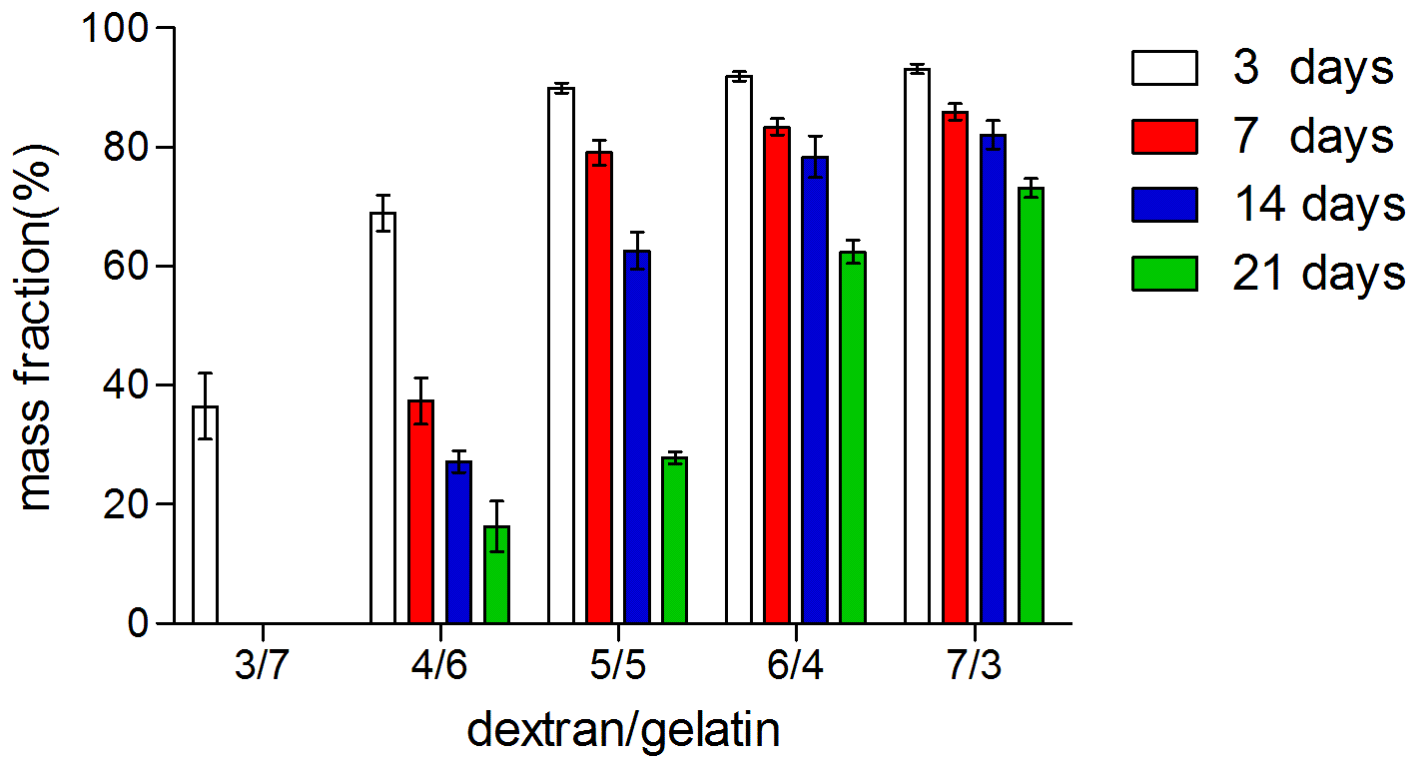
The degradation of dextran/gelatin hydrogels with different volume ratio.

The degradation of five composite hydrogels was monitored as by incubating in PBS at 37°C, as shown in Figure 6. Apparently, the ratio of dextran and gelatin has great impact on the weight loss of samples. Concretely, the 3/7 dextran/gelatin hydrogel exhibited the fastest degradation rate and totally degraded after 7 days. By contrast, 4/6 and 5/5 groups showed a more controllable degradation rate due to higher crosslinking density. Since gelatin can be easily solubilized in aqueous environment, groups of 6/4 and 7/3 with an incline of gelatin have the strongest resistance to degradation that they could still hold more than 50% of the initial weight after three weeks. Generally, it takes at least 14 days for the stem cells seeded in hydrogels to generate extracellular matrices for epidermal formation. So the results indicate that the degradation rate of the dextran/gelatin hydrogels could be modulated by altering the dextran content of hydrogel to match epidermal tissue formation.

#### 4. Mechanical properties

**Figure 7:**
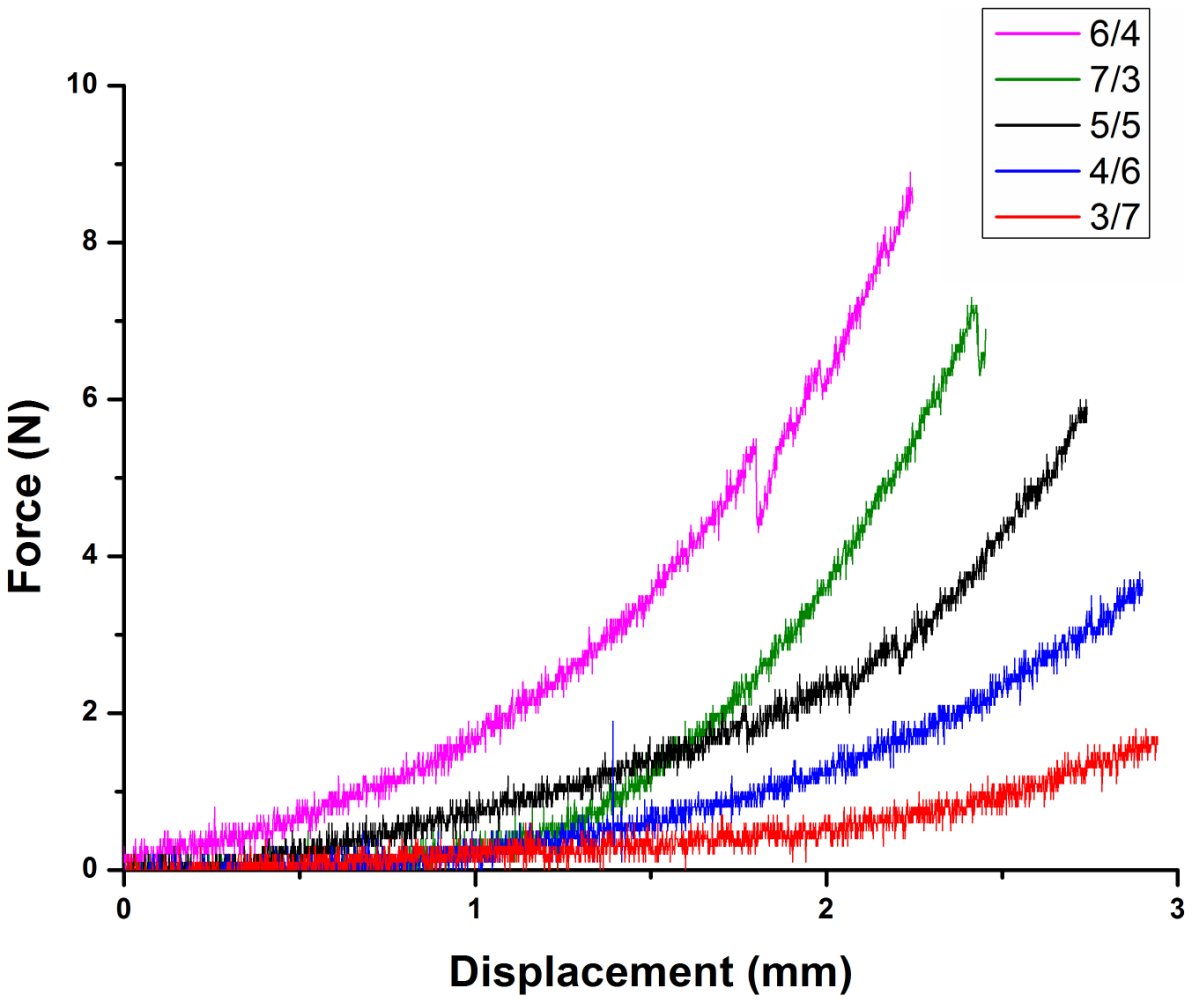
Force-displacement curves for all the compositions of dextran/gelatin hydrogels.

**Figure 8:**
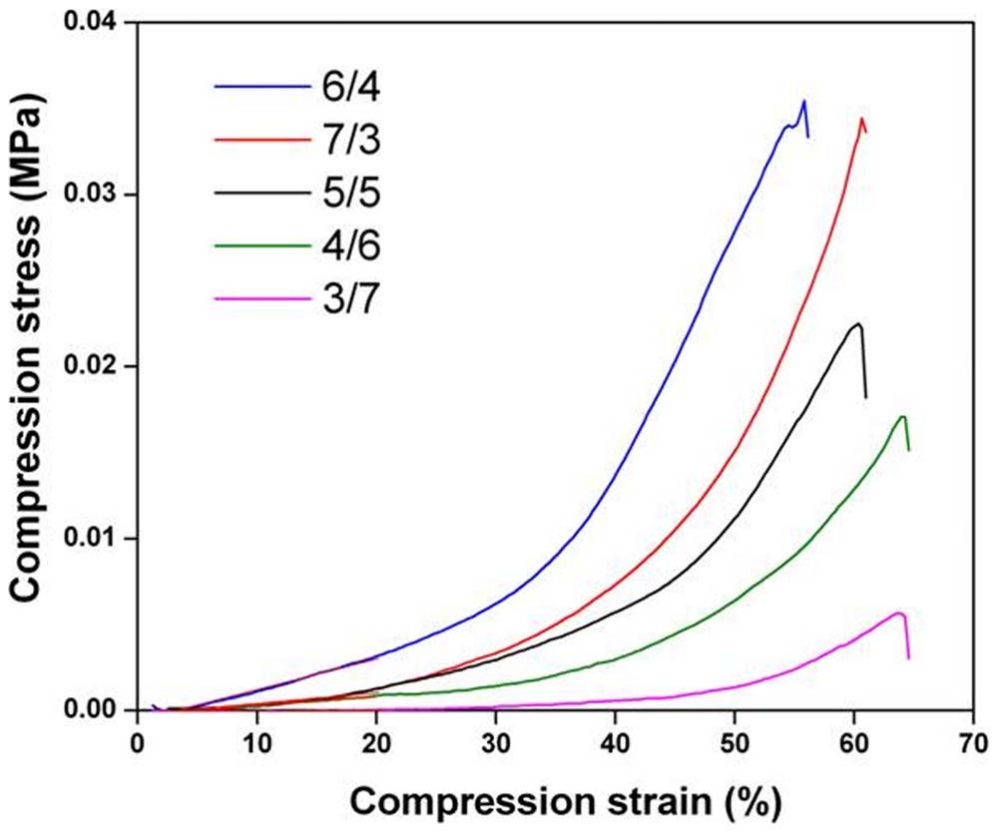
Stress-strain curves for all the compositions of dextran/gelatin hydrogels.

The effects of the difference ratio of dextran and gelatin on the mechanical properties were also evaluated. All mechanical tests were carried out at 37°C with fully hydrated hydrogel samples which were free from any physical imperfection. Figure 7 shows force-displacement curve for all the compositions. Compressive force corresponding to the same displacement value showed an increasing trend with an increase in dextran amount (from 3/7 to 5/5). The effect may be explained on the basis of formation of denser and condensed networks, which are rigid and thus require more stress to compress. Also, a representative compression curve is shown in Figure 8. As seen in the figure, all of the five groups show linear elastic behavior at low stress, non-linear switching strain at intermediate stress and linear elastic behavior at high stress. The modulus increases as the strain increases. This causes the stress at 50%–60% strain to be significantly larger than the stress at 10%–50% strain. The 6/4, 7/3, 5/5 dextran/gelatin have demonstrated the higher initial modulus than 4/6 and 3/7 hydrogel, which can be ascribed to the prefect crosslinking density and limited swelling property. The 3/7 and 4/6 dextran/gelatin hydrogels showed the worse mechanical property due to their great swollen condition. It can also be observed that the fracture strain of hydrogels increased slightly with the increase of gelatin content. The 3/7 dextran/gelatin hydrogel underwent higher deformation before failure than others although it failed at a lower stress. In general, all hydrogels deformed much less than 70%. This can be explained on the basis of the large pore size and high water content of the hydrogel. The imbibed water molecules migrate from the regions under load towards the unloaded regions thus resulting in deformation of the hydrogels as observed. For present purposes, the skin can be approximated as a bilayer, consisting of the epidermis (modulus, 140 to 600 kPa; thickness, 0.05 to 1.5 mm) and the dermis (modulus, 2 to 80 kPa; thickness, 0.3 to 3 mm) [23]–[24]. For the trends analyzed, the dextran/gelatin was suitable as the dermis substitute since the compressive modulus is close to the clinical range of modulus that will be expected for a material placed under the epidermis.

### Biocompatibility of the samples

#### 1. Cell viability assay of electrospun PLCL/Poloxamer membranes

**Figure 9:**
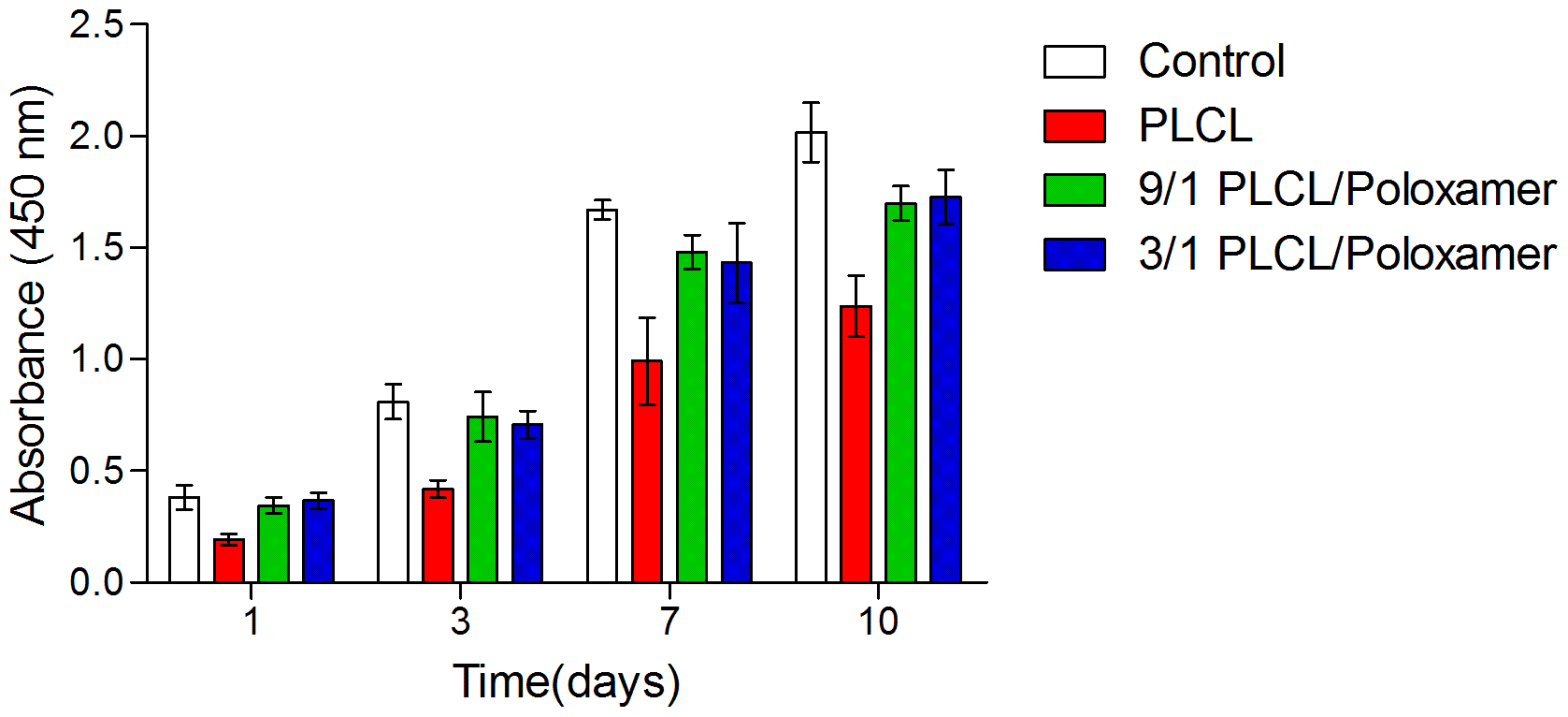
The proliferation of adipose-derived stem cells cultured on electrospun PLCL and PLCL/Poloxamer nanofibers in the CCK-8 assay: the absorbance of these medium with CCK-8 was read at 450 nm.

Adipose derived stem cells were used to determine the ability of the PLCL/Poloxamer membranes to support cell viability and proliferation. ADSCs were seeded onto various electrospun scaffolds and cultured. At different time points (1, 3, 7 and 10 days) the viability of ADSCs was determined by CCK-8 test and the values of absorbance at 450 nm were showed in Figure 9. The statistical analysis revealed that ADSCs had an increased metabolic activity on the electrospun PLCL/Poloxamer membranes with the increase of culture time, indicating the PLCL/Poloxamer membranes were able to support cell proliferation. And after 1, 3, 7 and 10 days in vitro culture, There was no significant difference in cell viability between the 9/1 group and the 3/1 group, while cell viability in PLCL/Poloxamer groups are significantly greater than PLCL group. The result may be explained on the basis of high hydrophilicity of PLCL/Poloxamer nanofibers, which facilitates the diffusion of tissue fluid and nutrients to support cell viability and proliferation. With the introduction of poloxamer, greater hydrophilic properties resulted in smaller contact angle as observed through water contact angle assay. The surface wettability is an important factor governing oxygen and nutrient permeability since the tissue fluid diffuses easily throughout the surface of membranes with high hydrophilicity.

#### 2. Cell viability assay of dextran/gelatin hydrogels

**Figure 10:**
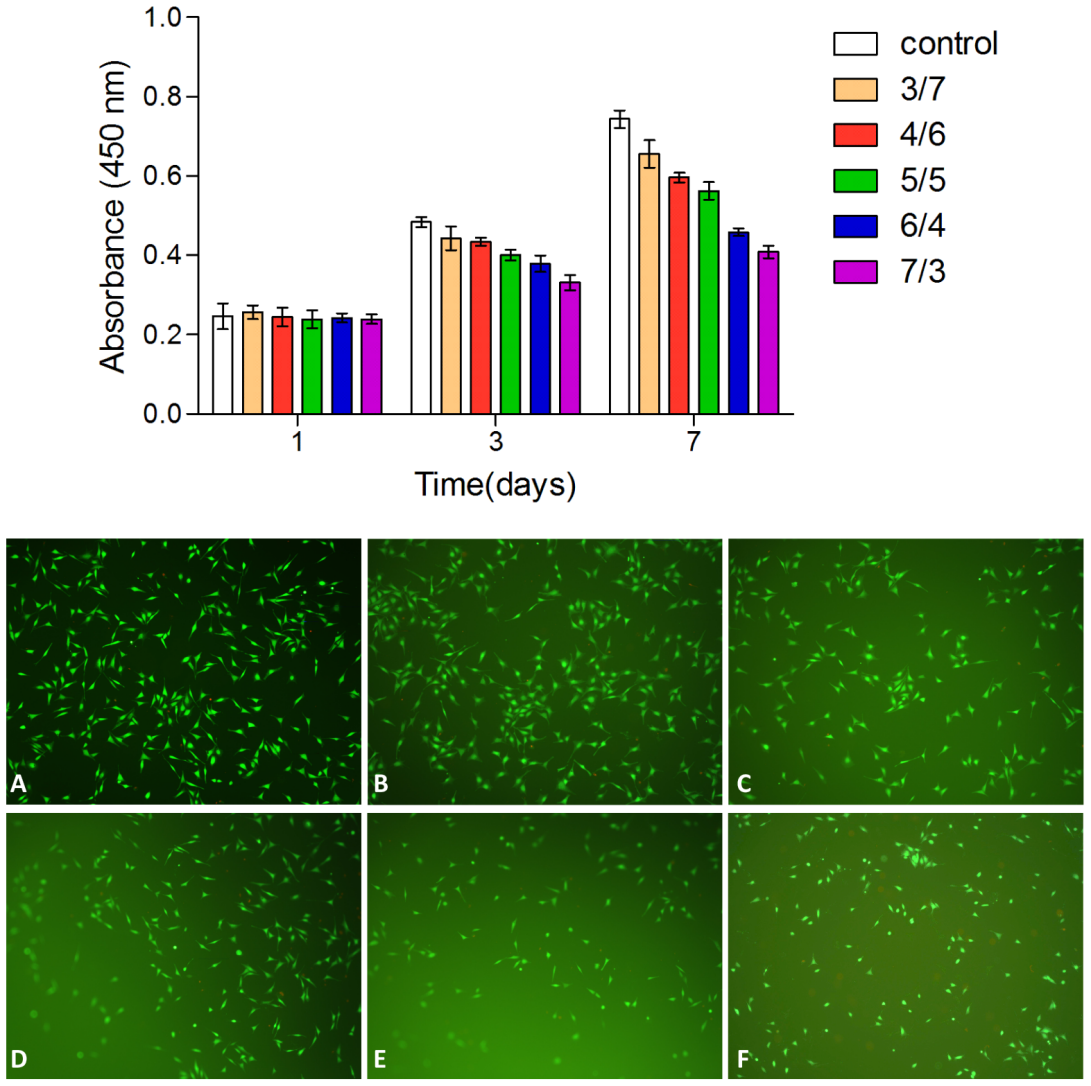
The proliferation of cells cultured on dextran/gelatin hydrogels in the CCK-8 assay: the absorbance of these medium with CCK-8 was read at 450 nm. Cells cultured on the hydrogels were stained with Live/Dead staining solution (A: tissue culture plate; B: 3/7; C: 4/6; D: 5/5; E: 6/4; F: 7/3).

Mouse pre-osteoblast cells were used to demonstrate the ability of the dextran/gelatin hydrogels to support cell proliferation since the dextran/gelatin hydrogels mimic natural extracellular matrix (ECM) of the dermis, which supports the extensive vasculature. The CCK-8 Assay was implemented to quantitatively assess cell viability of cells cultured on the hydrogels after 1, 3 and 7 days. As shown in Figure 10, there was a significant effect of gelatin concentration (p<0.05) and culture time (p<0.05) on cell viability. The statistical analysis revealed that cells had an increased metabolic activity on the hydrogels with the increase of incorporated gelatin. After 3 days culture, cells cultured on the hydrogels were stained with Live/Dead staining solution. In the fluorescence microscope it is showed that the hydrogel became a little adverse to cell responses such as the attachment, spreading and proliferation of cells with the ratio of dextran/gelatin change from 3/7 to 7/3. One possible explanation is that the side effect caused by the aldehyde groups in oxidized dextran hampered the adhesion of cells on matrix surface. After 7 days in vitro culture, cells on the surface of hydrogels remained viable and proliferative compared with the day 1 and 3 group, indicating the good cytocompatibility of hydrogels. These results implied that dextran/gelatin hydrogels might be good scaffold materials which have excellent biocompatibility.

#### 3. Cytotoxicity assay of bilayer scaffold

**Figure 11:**
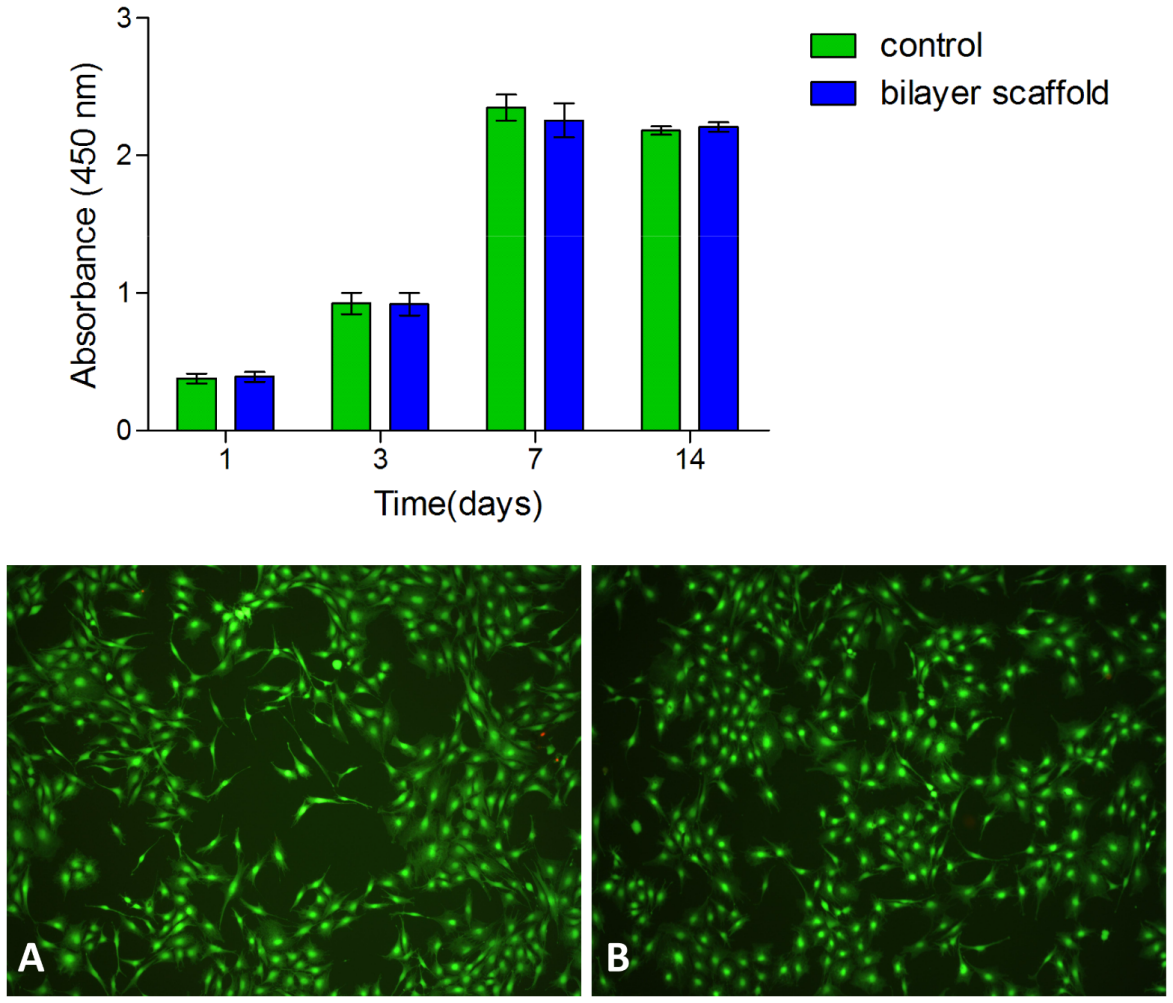
The proliferation of cells co-cultured with the bilayer scaffold in the CCK-8 assay. Cells were stained with Live/Dead staining solution (A: tissue culture plate; B: bilayer scaffold).

In addition to examining the two biomaterials that will comprise the bilayer scaffold, cytotoxicity assay data was also obtained on the constructed bilayer scaffold to determine if the bilayer composition affected the final result. Figure 11 shows the cell viability cocultured with bilayer scaffold compared to TCP. There was no statistically significant difference between the bilayer scaffold group and control group (p>0.05), indicating that the bilayer scaffold has no toxicity on cell viability. The statistical analysis revealed that cells had an increased metabolic activity with the increase of culture time. After 14 days culture, cells cocultured with or without bilayer scaffold were also stained with Live/Dead staining solution. In the fluorescence microscope the live and dead cells exhibit green-fluorescent and red-fluorescent. There was no visual difference between control group and bilayer scaffold group. The cells in two groups reached complete confluence and had an organized arrangement. All of them exhibited green-fluorescent morphology. Cells cocultured with bilayer scaffold remained viable and proliferative. These results implied that the degradation products derived from bilayer scaffold did not inhibit key metabolic pathways of cells.

## Discussion

In this study, we analysed the characteristics of electrospun PLCL/poloxamer nanofibers and dextran/gelatin hydrogels with the goal of fabricating a bilayer scaffold for skin tissue engineering applications. Electrospun nanofibers have received more and more attention to be used as tissue engineering scaffolds since they have the nanofibrous porous structure, which can mimic the native Extracellular Matrix (ECM) [25]–[28]. Utilizing electrospinning technology, PLCL and poloxamer can be processed into nanofiber membranes. PLCL is a synthetic biodegradable copolymer which is approved by FDA for both wound closure and orthopedic applications [29]–[30]. However, the hydrophobic property has restricted its applications. Poloxamer, which was also approved by FDA for human use, consists of hydrophilic poly(ethylene oxide) (PEO) and hydrophobic poly(propylene oxide) (PPO) blocks arranged in tri-block structure: PEO-PPO-PEO. Due to its amphiphilic character poloxamer displays surfactant properties including ability to interact with the surfaces of hydrophobic membranes [31]. In this study, poloxamer was used as a hydrophilic additive to PLCL membranes. The water contact angle of PLCL/poloxamer membranes decreased with the increase of incorporated poloxamer, indicating the addition of poloxamer improved the hydrophilicity. This may be provided by pore surface exposure of the hydrophilic PEO chains in poloxamer molecules entrapped within the PLCL/poloxamer membranes.

PLCL and poloxamer was blended together to be electrospun into nanofibers. The mechanical properties and biocompatibility of the obtained nanofibers were investigated. Pure PLCL nanofibers had the breaking strength of 7.23 MPa and elongation at break of 158%. When a small amount poloxamer was added in (content of 10%), the PLCL/Poloxamer nanofibers reached the higher tensile strength of 9.37 MPa, and it still keep the elasticity with the elongation at break of 187%. But with the further increasing of poloxamer content the mechanical properties of PLCL/poloxamer nanofibers decreased. Tensile strength and modulus of PLCL/poloxamer nanofiber membranes lie within the range of values suitable for human skin [32], providing these scaffolds with good resilience and compliance to movement as a skin graft.

Cell viability studies with adipose-derived stem cells demonstrated that PLCL/poloxamer nanofibers significantly promoted cell proliferation in comparison with PLCL nanofibers, especially when the weight ratio of PLCL to poloxamer was 9∶1. PLCL/poloxamer nanofibers showed better mechanical properties and biocompatibility than PLCL. One possible explanation is that the introduction of hydrophilic Poloxamer improves the electrospinnability thus forming hydrophilic nanofibers with interconnected and solider structure which facilitates the diffusion of tissue fluid and nutrients to support cell viability and proliferation. So that, the best way to get tissue engineering scaffolds with both excellent mechanical properties and biocompatibility is to combine two distinct materials together to form the blend nanofibers via electrospinning technology.

Native skin is composed of two layers with distinct qualities. One of the most used, commercially-available skin substitute is Integra [33], which is a bilayer scaffold composed of a silicone upper layer and a collagen-glycosaminoglycan porous sublayer. Thus, the bilayer scaffold composed of PLCL/poloxamer nanofiber upper layer and dextran/gelatin hydrogel sublayer should more accurately mimic the physical structure of the normal skin. The two separate layers have distinct functions which help the bilayer scaffold adapt to the complex environment of wound healing. The upper, PLCL/poloxamer nanofiber layer serves as a protective barrier against the outside world and integrates with the host skin tissue. The tensile strength and hydrophilic property provides good resilience and compliance to movement as a skin graft. The lower, dextran/gelatin hydrogel layer provide a highly swollen three-dimensional environment similar to soft tissues and fills in the hypodermis defect area. The swollen and biodegradable property allows for the ingrowth of surrounding native stem cells while maintaining significant amounts of tissue fluid on the wound bed and promoting diffusion of nutrients and cellular waste through the elastic networks.

Hydrogels are prepared by swelling cross-linked structures in water or biological fluids. Methods of preparing the hydrogel networks include chemical cross-linking(Schiff-based reaction, click reaction), environmentally or physiologically responsive cross-linking(self-assembly, thermosensitivity, pH-sensitivity) and photopolymerization [34]–[36]. Injectable hydrogels have gained widespread applications as three-dimensional tissue engineering scaffolds due to their advantages of taking the shape of a cavity and providing a good fit or interface between the hydrogel and host tissue. Moreover, various therapeutic molecules and even cells can be incorporated by simply mixing with the precursor solution prior to injection [37]–[38]. Dextran/gelatin hydrogel was fabricated through Schiff-based reaction between aldehyde groups and amino groups without the addition of a chemical crosslinking agent. Dextran and gelatin offer the advantage of being very similar to macromolecular substances of extracellular matrix and complete degradation by enzymes in vivo. The proliferation phase of the wound healing process is said to be about 3 weeks [39]. Therefore, a scaffold intended for temporary skin replacement should not completely disintegrate before this time to be able to perform its template function. Except dextran/gelatin hydrogel with the ratio of 3/7, other hydrogels meet this criterion and can support the proliferation of cells to repair the wound in skin tissue engineering. In respect of biocompatibility, with the ratio of dextran/gelatin change from 3/7 to 7/3, the matrix became adverse to cell viability and proliferation. So the bilayer scaffold consisting of 9/1 PLCL/poloxamer nanofiber upper layer and 5/5 dextran/gelatin hydrogel sublayer should be suitable to mimic the physical structure of the normal skin. And the results demonstrated that the bilayer scaffold has shown favorable in vitro biocompatibility as an acellular scaffold aimed to aid wound healing.

## Conclusions

In the present study, we prepared two different biomaterials and investigated the characteristics to fabricate a bilayer scaffold for skin tissue engineering applications. A bilayer design was conceived for an artificial skin substitute where distinct qualities of the two layers can be combined to enhance wound healing. The electrospun PLCL/Poloxamer nanofibers (9/1) displayed the optimal mechanical strength and biocompatible properties. The dextran/gelatin hydrogel (5/5) is a fast in situ forming scaffold that can support cell viability while possessing critical physical properties (mechanical strength and degradation) required for a skin tissue scaffold. The proposed combination of these two biomaterials can open more possibilities for wound treatment and rehabilitation as one system may not be sufficient to answer the complex environment in wound treatment and skin regeneration. This work provides a strategy for the design and fabrication of nanofiber-hydrogel bilayer scaffolds mimicking the structure of the normal skin for wound repair.
